# Aging Trajectories

**DOI:** 10.1093/geroni/igaf122.160

**Published:** 2025-12-31

## Abstract

These research presentations provide a research perspective on the trajectories of aging.

